# Dysregulated Cytokine Production by Dendritic Cells Modulates B Cell Responses in the NZM2410 Mouse Model of Lupus

**DOI:** 10.1371/journal.pone.0102151

**Published:** 2014-08-05

**Authors:** Allison Sang, Ying-Yi Zheng, Yiming Yin, Igor Dozmorov, Hao Li, Hui-Chen Hsu, John D. Mountz, Laurence Morel

**Affiliations:** 1 Department of Pathology, Immunology, and Laboratory Medicine, University of Florida, Gainesville, Florida, United States of America; 2 Department of Immunology, University of Texas Southwestern Medical Center, Dallas, Texas, United States of America; 3 Clinical Immunology and Rheumatology, Department of Medicine, University of Alabama at Birmingham, Birmingham, Alabama, United States of America; University of British Columbia, Canada

## Abstract

The breakdown in tolerance of autoreactive B cells in the lupus-prone NZM2410-derived B6.*Sle1.Sle2.Sle3* (TC) mice results in the secretion of autoantibodies. TC dendritic cells (DCs) enhance B cell proliferation and antibody secretion in a cytokine-dependent manner. However, the specific cytokine milieu by which TC DCs activate B cells was not known. In this study, we compared TC and C57BL/6 (B6) control for the distribution of DC subsets and for their production of cytokines affecting B cell responses. We show that TC DCs enhanced B cell proliferation through the production of IL-6 and IFN-γ, while antibody secretion was only dependent on IL-6. Pre-disease TC mice showed an expanded PDCA1^+^ cells prior to disease onset that was localized to the marginal zone and further expanded with age. The presence of PDCA1^+^ cells in the marginal zone correlated with a Type I Interferon (IFN) signature in marginal zone B cells, and this response was higher in TC than B6 mice. *In vivo* administration of anti-chromatin immune complexes upregulated IL-6 and IFN-γ production by splenic DCs from TC but not B6 mice. The production of BAFF and APRIL was decreased upon TC DC stimulation both *in vitro* and *in vivo*, indicating that these B cell survival factors do not play a role in B cell modulation by TC DCs. Finally, TC B cells were defective at downregulating IL-6 expression in response to anti-inflammatory apoptotic cell exposure. Overall, these results show that the TC autoimmune genetic background induces the production of B cell-modulating inflammatory cytokines by DCs, which are regulated by the microenvironment as well as the interplay between DC.

## Introduction

Systemic lupus erythematosus (SLE) is an autoimmune disease characterized by the loss of tolerance to self-antigens by B cells, resulting in the production of pathogenic autoantibodies (autoAbs). Dendritic cells (DC) can be classified into two main categories, classical DCs (cDC) and plasmacytoid DCs (pDC). Both pDCs and cDCs promote autoAb secretion by B cells. pDCs produce type I IFN in response to apoptotic cell autoantigens, and cDCs activated either by type I IFN or T cells secrete IL-6. Furthermore, both type I IFN and IL-6 can contribute directly or indirectly to autoAb production [1]. We have used the NZM2410-derived B6.*Sle1.Sle2.Sle3* (TC) lupus-prone mouse to investigate how DCs contribute to B cell dysfunction. TC mice are C57BL/6 (B6) congenic mice that express the three lupus susceptibility loci (*Sle1*, *Sle2*, *Sle3*) that are necessary and sufficient to induce a full clinical disease similar to that of NZM2410. The TC autoimmune phenotypes include early pre-disease lymphocyte activation, the production of high titers of anti-dsDNA IgG by 5 month of age, and lupus nephritis by 7 month of age [2].

We have previously shown that anti-CD40 stimulated bone-marrow derived DCs (BMDC) from TC mice secreted high levels of IL-6 and induced B cell proliferation and Ab production [3]. IL-6 is an essential cytokine for B cell proliferation and plasma cell differentiation [4], [5]. In SLE patients, IL-6 levels correlate with disease activity [6], [7]. Blocking IL-6 signaling in murine models of lupus ameliorated disease and suppressed the production of anti-dsDNA autoAbs [8], [9]. However, the inability of IL-6 antagonists to block DC-mediated B cell proliferation indicates that additional cytokines produced by TC DCs regulate B cell functions.

pDCs selectively express TLR7 and TLR9 which recognize RNA or DNA [10]. The activation of pDCs by apoptotic cell debris results in the production of large amounts of type I IFNs that have pleiotropic effects on B cells, including enhanced activation, survival, Ab and cytokine secretion [11]. A type I IFN signature has been recognized as a hallmark of lupus pathogenesis [12], and a recent study has identified a type I IFN signature in both cDCs and pDCs from TC mice that preceded disease onset, suggesting a causative role in pathogenesis [13].

Depletion of both cDCs and pDCs ameliorated lupus pathogenesis in the MRL/*lp*r model, including a decreased plasmablast numbers and autoAb production, suggesting a direct effect on B cells [14]. DCs can contribute to B cell-related pathogenesis both by the production of inflammatory cytokines and B cell survival factors. Cell-to-cell contact between activated DCs and B cells was not required to induce proliferation, which further confirmed the importance of DC-derived cytokines in lupus pathology [3]. Since there is no correlation between elevated DC numbers and autoimmunity [15], functional differences, including increased production of inflammatory cytokines, are most likely responsible for the role DCs play in lupus. The expression of CD86 is elevated on DCs from SLE patients, indicating that DCs have an activated phenotype during active disease [16]. Recent work has shown that DC-specific ablation of *Shp1*, a phosphatase highly expressed by DCs, led to a lupus-like phenotype with significantly elevated secretion of inflammatory cytokines by DCs [17]. Overall, these studies suggest that defects in DC regulation create an environment that fosters the activation of self-reactive B cells into Ab-producing cells.

In this study, we explore the effects of DCs from lupus-prone TC mice on B cell function. Our results show that anti-CD40 activated BMDCs from TC lupus-prone mice induce a greater B cell proliferation in an IL-6 and IFN-γ dependent manner. In addition, IL-6, but not IFN-γ produced by DCs, enhances the secretion of IgM by B cells. Confirming these results, splenic DCs from TC mice produce elevated levels of IL-6 and IFN-γ in the presence of anti-chromatin ICs. Total splenic DCs from TC mice have an expanded pDC population that is largely concentrated in the marginal zone (MZ). This expansion correlated with a type I IFN signature in TC MZ B cells, suggesting an interaction between pDCs and MZ B cells in the lupus mice. Finally, MZ B cells from TC mice maintained a higher IL-6 secretion in response to anti-inflammatory apoptotic cells than MZ B cells from B6 mice. Overall, these results demonstrate that the dysregulation of cytokine networks in DCs from lupus mice, in which IL-6 and both type I and II IFNs play a major role, contribute to the activation of pathogenic B cells.

## Materials and Methods

### Mice

The B6.NZM-*Sle1^NZM2410/Aeg^ Sle2^NZM2410/Aeg^ Sle3^NZM2410/Aeg^*/LmoJ (TC in this paper) congenic strain has been previously described [18]. Age-matched C57BL/6J (B6) mice were used for all experiments. Only female mice were used in this study at the age indicated for each experiment. All mice were bred and maintained at the University of Florida in specific pathogen-free conditions.

### Ethic statement

Experiments using mice reported here were approved by the Institutional Animal Care and Use Committee of the University of Florida (UF #201303860).

### Flow Cytometry

Single cell suspensions of splenocytes were treated with 155 mM of NH_4_Cl for 5 min to lyse red blood cells and were passed through a pre-separation filter (Miltenyi Biotec) to remove debris. Cells were blocked on ice for 30 min with 10% rabbit serum and anti-CD16/32 (2.4G2) in staining buffer (2.5% FBS, 0.05% sodium azide in PBS). Cells were then stained for 30 min on ice with pre-determined amounts of the following fluorophore-conjugated or biotinylated Abs: CD21 (4E3), CD23 (B3B4), IgM (II/41), CD11c (HL3), CD8 (53-6.7), DCIR2 (33D1), DEC205 (205yekta), PDCA-1 (927), IFNγ (XMG1.2), all purchased from either eBiosciences or BD Biosciences. Dead cells were gated out with the Fixable Viability Dye eFluor 780. Intracellular staining for IFN-γ was performed on cells fixed and permeabilized with eBiosciences reagents. Stained cells were processed on a CyAn flow cytometer (Beckman Coulter) and at least 100,000 cells were acquired per sample. Flow cytometry data sets were analyzed with the FCS Express software (De Novo).

### Generation of BMDCs and BMDC/B Cell Co-Cultures

Bone marrow (BM)-derived DCs (BMDCs) were differentiated with 10 ng/ml GM-CSF (Peprotech) and 5 ng/ml IL-4 (Peprotech), then purified with anti-CD11c beads (Miltenyi Biotec), as previously described [3]. CD43^−^ B cells were isolated from B6 splenocytes by negative selection (Miltenyi Biotec). For BMDC-B cell co-cultures, 2×10^4^ BMDCs from either TC or B6 mice were cultured for 5 d with 10^5^ B cells and 10 ug/ml anti-CD40 Ab (1C10) (eBioscience) in duplicate as previously described [3]. The effect of cytokine inhibition on B cell proliferation was studied by adding 10 ug/ml of anti-IL-6 (MP5-20F3) (BD Biosciences) and/or anti-IFN-γ (XMG1.2) (eBioscience) to the co-cultures. To quantify cytokine secretion by activated BMDCs, 2×10^4^ BMDCs were cultured with 10 ug/ml anti-CD40 Ab for 24 h, after which RNA was extracted for qRT-PCR and gene array analyses, and supernatants were collected from the cultures for ELISA. IFN-γ was also quantified by intracellular flow cytometry, and BMDCs stimulated with either IL-12 (5 ng/ml) or IL-18 (20 ng/ml) were used as positive controls. Mice used in these experiments were 2–3 months of age.

### *In Vivo* Cytokine Production

Two month old mice were first injected i.p. with 250 ul of pristane (Sigma) on d0 and d7. On d10, they were injected with 10^7^ cells from the PL2-8 hybridoma (anti-chromatin IgG2b) [19] or from the C4010 hybridoma (anti-TNP IgG2a^b^) [20], or with PBS, then sacrificed on d17. DCs from mice that received the hybridoma cells or controls were isolated from collagenase (Roche) -digested spleens by positive selection with anti-CD11c magnetic beads as previously described [21].

### Cytokine and Gene Expression Quantification

Gene expression was quantified by qPCR from RNA extracted from BMDCs, splenic DCs or from sorted MZ/FO B cells using Sybr Green (Applied Biosystems) as previously described [22]. *Gapdh* was used as internal control. The results were normalized to the average unstimulated or 2 month old B6 values. The primers used are listed in Table 1. In addition, a Taqman Gene Expression Assay (Applied Biosystems) was used to measure *Irf7* (Mm00516788_m1) expression relative to *Ppia* (Mm02342429_g1) endogenous control. ELISA kits were used to quantify IL-6, IL-10, IFN-γ (BD Biosciences), and BAFF (R&D Systems) from the culture supernatants. Additional cytokines from culture supernatants were assessed using the Mouse Autoimmune Response Multi-Analyte ELISArray Kit (Qiagen), all according to the manufacturers' instructions. Microarray gene expression profiling was performed from B6 B cells cultured for 5 d with the supernatant of anti-CD40-activated BMDCs from either B6 or TC mice (N = 4 in each group), as previously described [3]. cDNAs from the B6 B cells was synthesized and labeled with the Ovation Biotin RNA Amplification and Labeling System (NuGEN Technologies, Inc.) before hybridization to Affymetrix Mouse Genome 430 2.0 arrays. The analysis was conducted as previously described [23]. Functional analysis of identified genes was performed with Ingenuity Pathway Analysis (IPA; Ingenuity Systems, Redwood City, CA). In this paper, we focused on the IFN-γ inducible genes that were differentially expressed between the B cells stimulated with supernatant from either TC or B6 BMDCs with at least a 2 fold difference and a p value≤0.01 for 2-tailed *t* tests.

**Table 1:** Primer sequences for qPCR.

Gene	Forward Primer	Reverse Primer
IL-6	ACCAGAGGAAATTTTCAATAGGC	TGATGCACTTGCAGAAAACA
IL-10	TGTCAAATTCATTCATGGCCT	ATCGATTTCTCCCCTGTGAA
IFN-γ	GAGCTCATTGAATGCTTGGC	GCGTCATTGAATCACACCTG
BAFF	TGCCTTGGAGGAGAAAGAGA	GGCAGTGTTTTGGGCATATT
APRIL	CAGTCCTGCATCTTGTTCCA	GCAGATAAATTCCAGTGTCCC
Blimp-1	GCCAACCAGGAACTTCTTGTGT	AGGATAAACCACCCGAGGGT
Isg-15	GAGCTAGAGCCTGCAGCAAT	TAAGACCGTCCTGGAGCACT
Mx1	GATCCGACTTCACTTCCAGATGG	CATCTCAGTGGTAGTCAACCC
Gapdh	AGCTTGTCATCAACGGGAAG	GTGGTTCACACCCATCACAA

### Confocal Imaging and Quantitation

Spleens from 2 and 7 month old B6 and TC mice were snap-frozen in Tissue TeK freezing medium (Fisher). Seven micrometer thick frozen sections were fixed to slides in ice-cold acetone for 15 min, air dried for 30 sec and blocked with 1.5% BSA in PBS for 30 min at room temperature. The sections were then stained for 30 min at room temperature in a humidified chamber with purified rat anti mouse PDCA-1 antibody (rat IgG2b; Miltenyi Biotec) and followed by Alexa 555–conjugated goat anti-rat IgG (Life Technologies) for another 30 min. Sections stained only with fluorescence labeled secondary antibody were used as control. All tissue sections were mounted in ProLong Gold Antifade Reagent (Life Technologies) and viewed with a Leica DM IRBE inverted Nomarski/epifluorescence microscope outfitted with Leica TCS NT laser confocal optics. Imaging quantitation was performed with MetaMorph 7.5, image analysis (Molecular Devices, Downingtown, PA, USA). The number of PDCA-1^+^ cells was computed for the whole splenic section as well as for the marginal zone.

### Apoptotic Cell Cultures

To generate apoptotic cells, thymocytes were cultured with 1 uM Dexamethasone (Sigma) for 4 h at 37°C. Staining with 7AAD and Annexin V (BD Biosciences) determined that this treatment typically resulted in 45% of Annexin V^+^ apoptotic thymocytes and in 1% of 7AAD^+^ Annexin V^-^ necrotic thymocytes. Marginal zone and follicular B cells were sorted from purified splenic CD43^−^ B cells as IgM^+^CD21^+^CD23^−^ for MZ B cells and IgM^+^CD21^−^CD23^+^ for FO B cells, using a FACS Aria-II cell sorter (BD Biosciences). Post-sorting purity of either subset was greater than 90%. The sorted MZ or FO B cells were co-cultured at a 1∶7 ratio with apoptotic thymocytes and 1 ug/ml CpG-B (Invivogen) for 3 d at 37°C. Cytokines were quantified from extracted RNA and culture supernatant by qPCR and ELISA, respectively.

### Statistical Analysis

Data analysis was performed with GraphPad Prism 6.0 software. Unless indicated, graphs show means and standard errors of the mean (SEM). Statistical significance between strains was determined by two-tailed Mann-Whitney tests or *t* tests (paired when appropriate) if the data was normally distributed. Multiple comparison test corrections were applied when needed. When indicated, results were normalized to average values for control B6 samples. Significance levels in figures were labeled as * for p<0.05, ** for p<0.01, and *** for p<0.001.

## Results

### BMDCs from TC lupus-prone mice enhance B cell proliferation through IL-6 and IFN-γ

**Figure 1:**
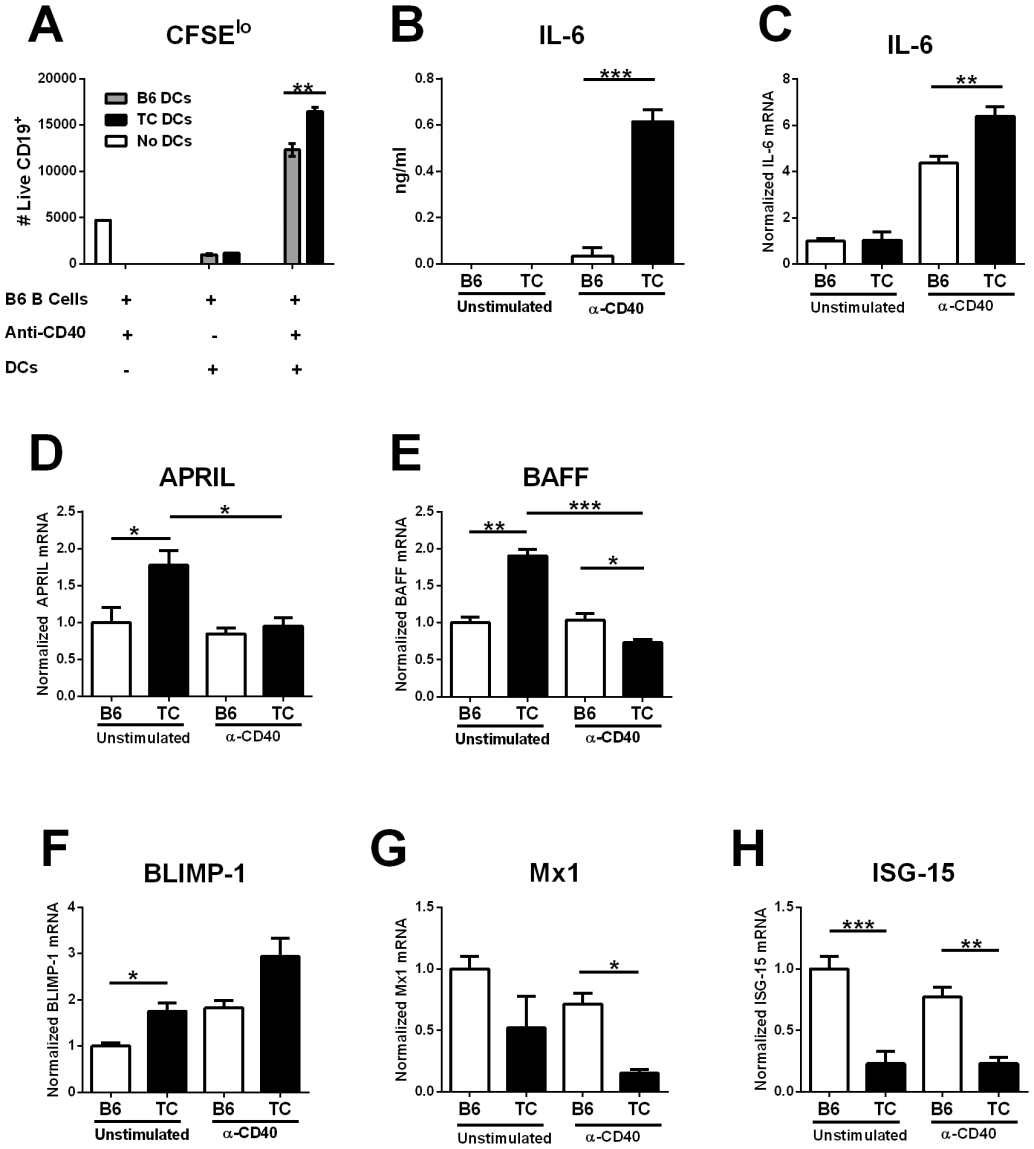
Anti-CD40 activated TC-derived BMDCs secrete high levels of IL-6. **A**. 10^5^ B6 B cells were co-cultured with 2×10^4^ TC or B6 BMDCs for 5 d in the presence of anti-CD40 Ab to activate the BMDCs. As controls, B cells were cultured alone with anti-CD40, and DCs were co-cultured with B cells in the absence of anti-CD40. B cell proliferation was measured as the number of live CD19^+^ CFSE^lo^ cells. Data are representative from two independent experiments. **B**. IL-6 production in the supernatant from 2×10^4^ BMDCs stimulated or not with anti-CD40 Ab for 24 h. **C-H**. IL-6, APRIL, BAFF, BLIMP-1, *Mx1*, and *Isg-15* gene expression in BMDCs stimulated or not with anti-CD40 Ab for 24 h. Message expression was normalized to *Gapdh* and expressed relative to the mean values for unstimulated B6 BMDCs for each gene. Significance levels of Bonferroni's multiple comparisons test are shown (N = 3 mice per strain per experiment).

To compare the effect of T-cell activated DCs on B cells between lupus-prone TC and B6 mice, we used co-cultures of anti-CD40 activated BMDCs from either strain and B6 B cells [3]. Without Flt3L, BMDCs have been shown to mainly correspond to cDCs [24]. Anti-CD40-activated BMDCs obtained from TC mice resulted in a greater number of live B6 B cells than B6 BMDCs *in vitro* (Fig. 1A), confirming our previous results [3]. No difference was observed in the number of dead cells (data not shown). As previously reported, CD40 stimulation in B cells in the absence of BMDCs resulted in similar low levels of proliferation in both strains, indicating that the primary target of anti-CD40 activation in the co-cultures are the DCs. In addition, unstimulated DCs from either strain were not able to support B cell proliferation. Since cell-to-cell contact was not required between TC DCs and B cells to enhance B cell proliferation [3], we compared cytokine secretion between unstimulated and anti-CD40 stimulated B6 and TC BMDCs. As reported previously [3], [25], anti-CD40 stimulated TC BMDCs secreted significantly more IL-6 than B6 BMDCs, and no IL-6 was secreted from unstimulated BMDCs in either strain (Fig. 1B). In addition, *Il6* message expression was higher in anti-CD40 stimulated TC than B6 BMDCs although no difference was seen in the absence of stimulation (Fig. 1C).

Quantification of B cell survival factors APRIL and BAFF showed that unstimulated TC BMDCs had higher expression than B6 BMDCs for both cytokines (Fig. 1D–E), which confirms previous studies reporting higher levels of BAFF in these mice [26]. Anti-CD40 stimulation significantly decreased APRIL and BAFF expression in TC BMDCs but not B6 BMDCs. BAFF secretion could not be detected in the culture supernatant of either stimulated or unstimulated DCs (data not shown). This suggests that the enhanced B cell proliferation mediated by anti-CD40 stimulated TC-BMDCs does not result from an increased B cell survival induced by either of these two cytokines. A recent study has shown that the transcriptional repressor Blimp-1 has a tolerogenic function in DCs, and Blimp-1-deficient DCs over-express IL-6, which lead to the development of lupus-like autoAbs [27]. However, Blimp-1 expression was increased in unstimulated and there was a trend for stimulated TC BMDCS as compared to B6 (Fig. 1F). This result demonstrates that the increased IL-6 production by activated TC DCs is not due to a decreased *Prdm1* (encoding for Blimp-1) expression. We also compared the expression of type 1 IFN inducible genes that have been reported to be increased in DCs from young TC mice [13]. Contrary to this previous study, however, we found that TC BMDCs express significantly lower levels of both *Mx1* and *Isg-15* than B6 BMDCs (Fig. 1G-H).

**Figure 2:**
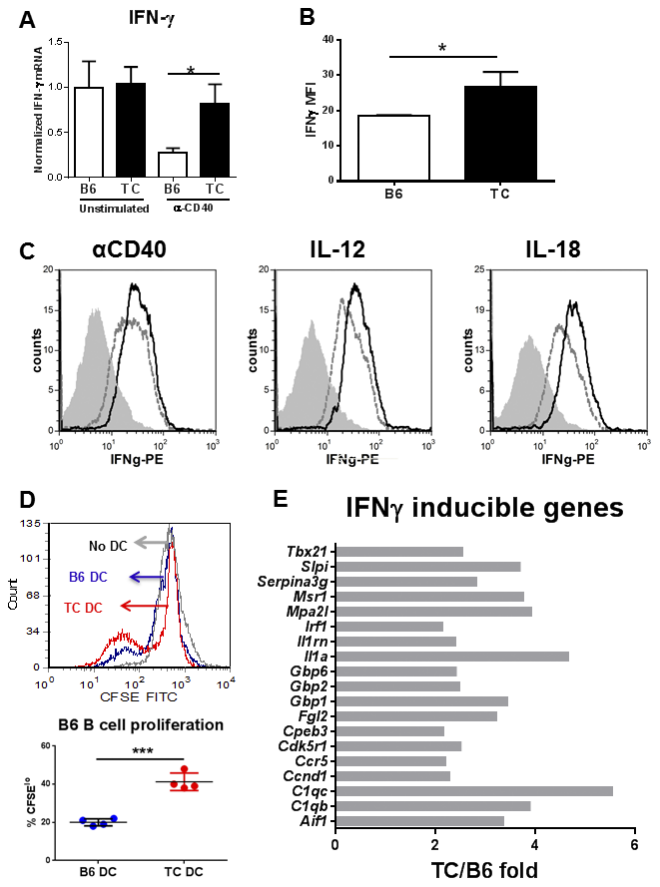
Anti-CD40 activated TC-derived BMDCs secrete IFN-γ. **A**. IFN-γ gene expression in BMDCs stimulated or not with anti-CD40 Ab for 24 h. Message expression was normalized to *Gapdh* and expressed relative to the mean values for unstimulated B6 BMDCs for each gene. (N = 7 per group). **B**. Intracellular IFN-γ expression expressed as mean fluorescence intensity (MFI) in BMDCs stimulated or not with anti-CD40 Ab for 24 h (N = 4 per strain). **C**. Representative FACS plots showing intracellular IFN-γ staining in CD11c^hi^ viability dye-negative anti-CD40 stimulated BMDCs (corresponding to the graph in B). The middle and right-side overlays shown BMDCs stimulated with IL-12p70 and IL-18 as controls. The solid black lines correspond to TC DCs and the broken lines correspond to B6 DCs. The filled gray histograms show the isotype control. **D**. B6 B cell proliferation measured as CSFE dilution in response to anti-CD40-activated TC BMDC (red) or B6 BMDC (blue) supernatant. The profile of B cell alone is shown as control. Representative FACS overlays and quantitation. **E**. Differential expression of IFN-γ-inducible genes in the B cells shown in **D**. The graph shows the TC over B6 fold expression for all the IFN-γ-inducible genes that are expressed as a p<0.01 different level between the B cells activated with the two different types on activated BMDCs.

IFN-γ expression was similar between unstimulated BMDCs from the two strains, but it was significantly higher in TC than B6 BMDCs after anti-CD40 stimulation (Fig. 2A). This difference of expression was confirmed by intracellular flow-cytometry (Fig. 2B), and anti-CD40 stimulation induced a similar IFN-γ production in TC BMDCs than either IL-12p70 or IL-18 (Fig. 2C), two cytokines that have been reported to induce IFN-γ production in DCs [28]. In addition, either IL-12 or IL-18 induced a higher production of IFN-γ by TC than B6 BMDCs (MFI: 30.51+3.18 vs. 21.47+.71 for IL-12; 27.24+2.32vs. 19.78+1.10 for IL-18; p<0.05). To determine the mechanisms by which TC DC-produced cytokines regulated B cell functions, we compared the gene expression in B6 B cells that had been stimulated with the supernatant of either B6 or TC anti-CD40-stimulated BMDCs. As previously shown [3], the TC-produced supernatant induced a greater percentage of B cells to proliferate than the B6-produced supernatant (Fig. 2D). The B cells stimulated by TC-produced supernatant showed a significant activation of the IFN-γ pathway (Fig. S1), with 19 differentially expressed IFN-γ inducible genes that were found at least a two-fold higher level than in B cells stimulated with B6-procuded supernatant (Fig. 2E). No IFN-γ inducible gene was found at a significantly lower level in B cells stimulated with TC-produced supernatant (data not shown). These results show that anti-CD40 stimulates IFN-γ production in TC DCs, which in turn activates a transcription program in B cells. Interestingly, the array analysis also showed an increase in *Il17a* message expression in B cells exposed to TC-produced supernatant (235.50+68.56 vs. 40.75+1.80, p = 0.03), as well as in the expression of genes induced by *Il17a* (Fig. S1). IL-6 is a major inducer of IL-17 expression in T cells [29], although IL-6- and RORγt-independent Il-17 programming can be induced by in B cells by *Trypanosoma* [30]. Our data suggest that IL-6 produced by activated TC BMDCs induce IL-17 expression in B cells. Overall, our results showed that IL-6 and IFN-γ are the two major cytokines that are over-expressed in T cell-activated TC BMDCs, and suggest that they may be responsible for the functional changes that we have described in TC DC-stimulated B cells.

**Figure 3:**
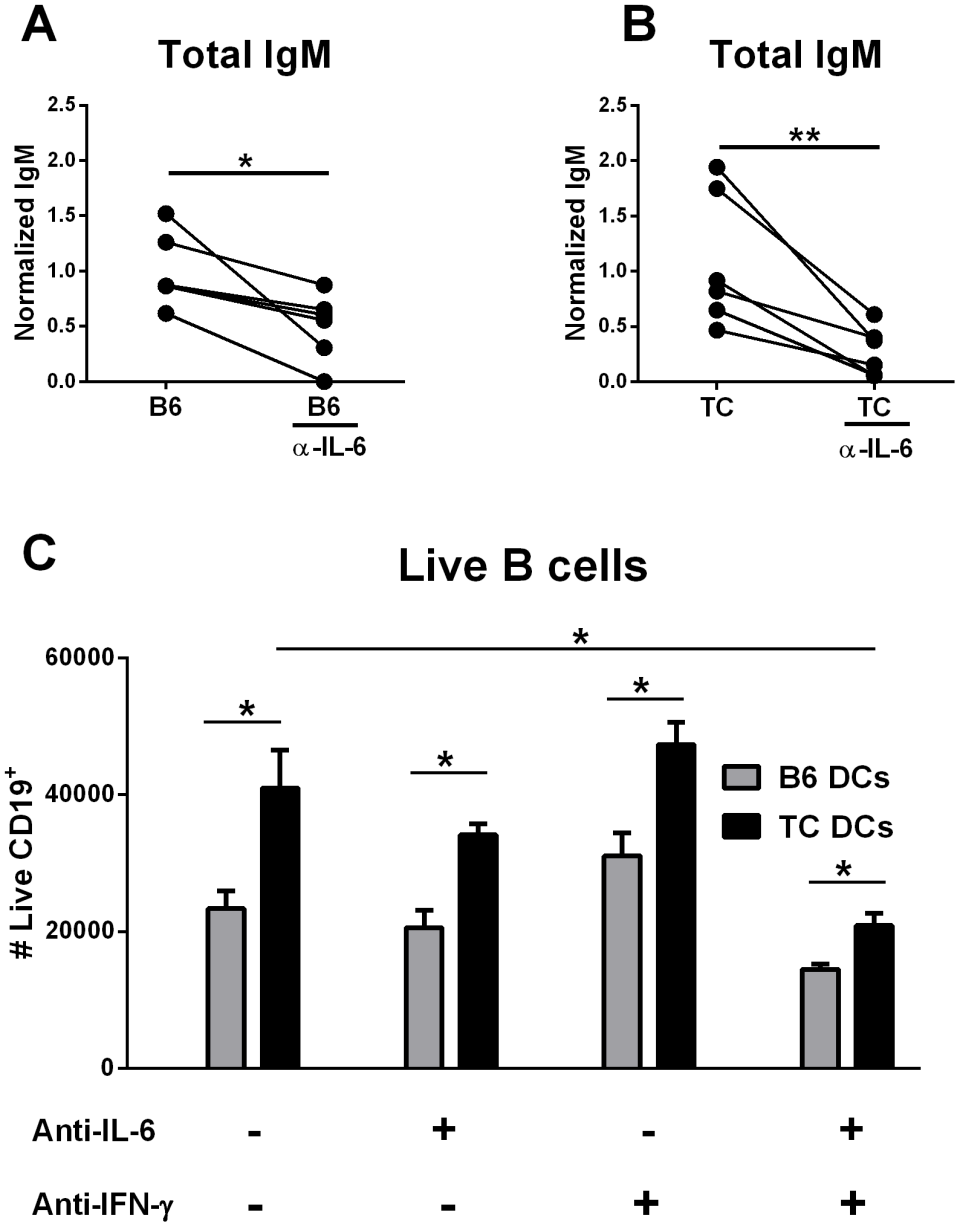
Enhanced B cell proliferation by TC BMDCs is IL-6 and IFN-γ dependent. 2×10^4^ BMDCs were co-cultured with 10^5^ B cells with anti-CD40 Ab for 5 d. Blocking Abs for IL-6 and/or IFN-γ were added to the co-culture. Blocking IL-6 significantly decreased IgM secretion in co-cultures with either B6 (**A**) or TC (**B**) BMDCs. The significant reduction of the B cell numbers in the TC BMDC co-cultures required the combined blocking of IL-6 and IFN-γ (**C**). Significance was determined by two-tailed paired tests.

IL-6 is a critical cytokine secreted by DCs that induces immunoglobulin production from B cells [31], [32]. Accordingly, blocking IL-6 in the DC/B cell co-cultures significantly decreased IgM production induced by either B6 or TC BMDCs (Fig. 3A and B). However, blocking of IL-6 was not sufficient to significantly reduced B cell proliferation induced by either B6 or TC BMDCs (Fig. 3C). Similarly, blocking IFN-γ, the other cytokine produced at higher levels by activated TC BMDCS has little effect on B cell proliferation (Fig. 3C), and had no effect on IgM secretion (data not shown). However, dual inhibition of IL-6 and IFN-γ significantly reduced B cell proliferation induced by both TC and B6 BMDCs, and reduced the effect of TC BMDCS to that of untreated B6 BMDCs. This significant reduction in B cell numbers obtained with the blocking antibodies did not result from increased cell death in the TC BMDC co-cultures as the number of dead B cells in the culture was equal in the B6 and TC BMDC co-cultures (data not shown). Therefore, TC DC-derived IL-6 and IFN-γ in combination increase B cell proliferation and, in addition, DC-derived IL-6 increases IgM production.

### Plasmacytoid DCs are expanded in TC mice at a young age

**Figure 4:**
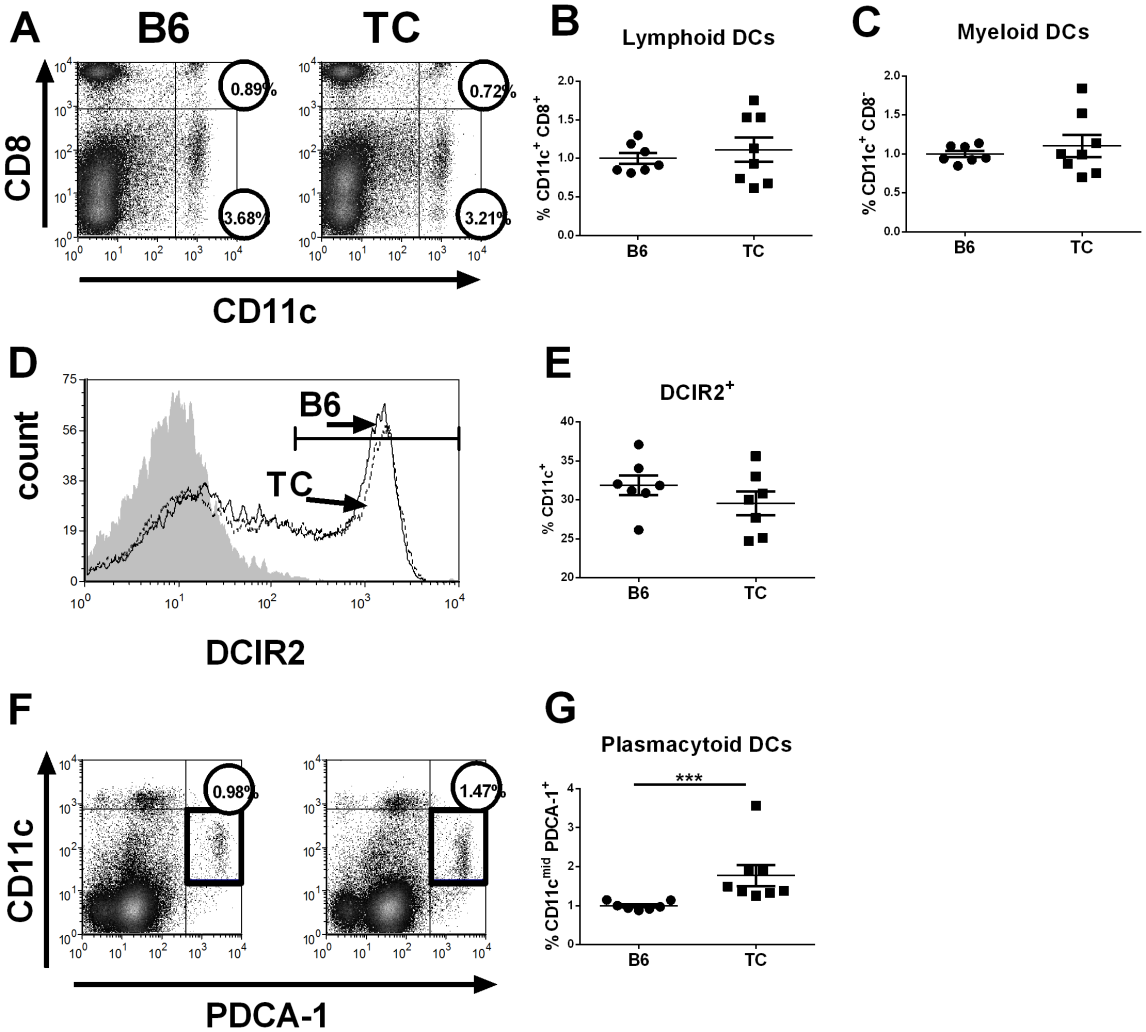
The plasmacytoid DC subset is expanded in young TC mice. Splenic DCs were isolated from 2-3 month old TC and B6 mice. **A**. Representative FACS plots showing the percentage of (**B**) myeloid DCs (CD11c^+^ CD8^−^) and (**C**) lymphoid DCs (CD11c^+^ CD8^+^). **D**. Representative histogram of DCIR2^+^ expression on CD11c^+^ splenic DCs in B6 (black line) and TC (dotted line) mice. The shaded histogram shows the isotype control and the horizontal line the gate for DCIR2^+^ DCs. The percentage of splenic CD11c^+^ DCs expressing DCIR2 in each strain is shown in (**E**)**. F**–**G**. Plasmacytoid DCs were identified as CD11c^mid^ PDCA-1^+^. Graphs show data from two experiments that were normalized to B6 means. Significance levels of two-tailed Mann-Whitney tests are shown.

We characterized splenic cDC and pDC subsets in young TC and B6 mice to assess whether the differences observed between B6 and TC BMDCs precede disease onset *in vivo*. The cDC subset can be subdivided into migratory CD8^-^ myeloid DCs and CD8^+^ resident lymphoid DCs [33], [34]. Two month old TC and B6 mice had similar percentages of both myeloid (CD11c^+^ CD8^-^) and lymphoid (CD11c^+^ CD8^+^) splenic DCs (Fig. 4A-C). Since previous results showed that aged TC mice have elevated numbers of myeloid DCs [25], this suggests that the expansion of that myeloid DCs is age-dependent and may be secondary to disease onset. Thus given that the BMDCs in our cultures are mainly cDCs, as pDCs require Flt3L for differentiation [24], elevated levels of IL-6 and IFN-γ are produced by activated TC cDCs that are expanded after disease onset. We also evaluated a subset of MZ-localized DCIR2^+^ DEC205^-^ DCs that have been shown to play a role in the activation of extrafollicular B cells [35]. However, young TC and B6 mice showed similar percentage of DCIR2^+^ (Fig. 4D-E), or DEC205^+^ splenic DCs (data not shown). This indicated that this recently described DC subset is not involved in the TC model.

**Figure 5:**
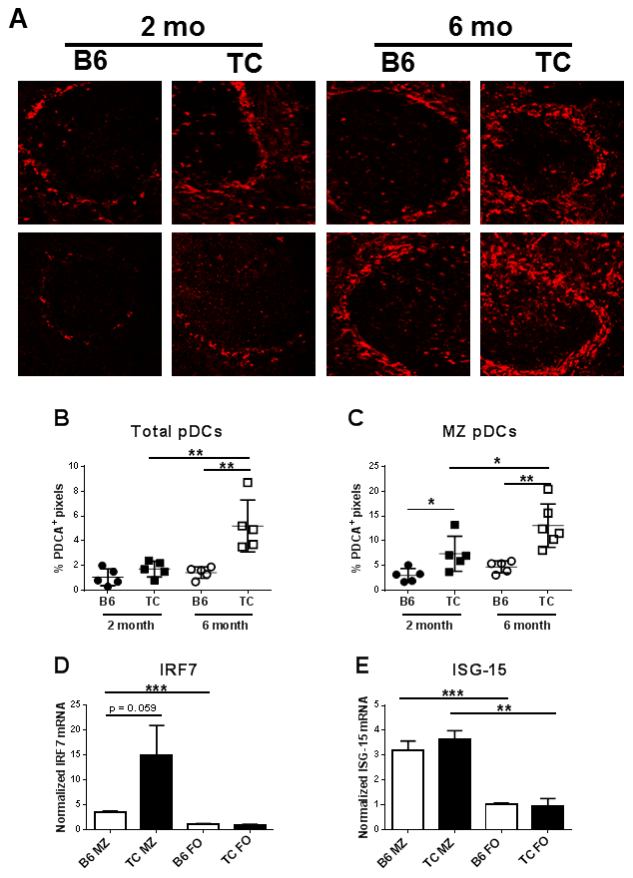
pDCs accumulation in the MZ of TC mice is correlated with a Type I IFN response in MZ B cells. **A**. Representative splenic sections stained with PDCA-PE showing localization of pDCs in representative 2 month or 6 month old B6 and TC mice. PDCA-1 staining is shown in red (Magnification at 20× dry objective with zooming at 1024*1024; The final magnification is approximately 14.47×20). The percentage of pDCs in total (**B**) and in the MZ (**C**) per splenic section. The location of the marginal zone was determined by the simultaneous presence of IgM^+^ B cells and SIGN-R1^+^ MZ macrophages (data not shown). Data includes 2–3 splenic sections per mouse from two mice per strain per age group. RNAs from sorted MZ or FO B cells cultured with CpG-B for 3 d were assayed for *Irf7* (**D**) and *Isg-15* (**E**) expression by qRT-PCR. Message expression was normalized to *Gapdh*. Significance levels of two-tailed t tests are shown.

Importantly, the percentage of CD11c^mid^ PDCA-1^+^ pDCs was significantly higher in young TC mice than in B6 (Fig. 4F, G). It is interesting that while the young B6 mice had a very consistent percentage of pDCs (about 1% splenocytes), the distribution of the TC values was much more variable (F = 51.39, p = 0.001), suggesting that this variable rate of expansion of pDCs may be related to the variation in disease onset that is typically observed in these mice [2]. The pDC expansion documented by flow cytometry was confirmed by histology, which showed that the abundance of pDCs in TC spleens was greater than in B6 spleens (Fig. 5A–C). Furthermore, PDCA-1 staining in the spleen showed that pDCs were largely confined to the MZ in both strains (Fig. 5A). The percentage of pDCs in the MZ was higher in TC mice both at 2 month (pre-disease) and at 6 month of age (ongoing autoimmune disease) than in B6 mice. Moreover, the MZ pDCs expanded with age in TC mice, but not in B6 mice (Fig. 5C).

pDCs are known for their capacity to secrete high levels of Type I IFNs [10]. TC splenic B cells express a Type I IFN signature prior to disease onset [13]. The preferential localization of pDCs in the MZ of TC mice suggested that the IFN response in B cells was dependent on their location. To test this hypothesis, RNA was extracted from sorted MZ or follicular (FO) B cells that were cultured with CpG-B, a TLR9 ligand with a full phosphorothioate backbone that strongly activates B cells but stimulates weakly IFN-α secretion. *Irf7* and *Isg-15* are two Type I IFN stimulated genes that are expressed at high levels in splenic B cells from young TC mice as compared to B6 [13]. Consistent with our finding that pDCs are largely located in the MZ, *Irf7* and *Isg-15* expression was higher in MZB than in FOB cells from both strains (Fig. 5D-E). In addition, *Irf7* expression tended to be higher (p = 0.059) in TC than in B6 MZ B cells (Fig. 5D), but *Isg-15* expression was similar between B6 and TC MZ B cells (Fig. 5E). Meanwhile, *Mx1* expression, a commonly used indicator of the Type I IFN signature, could not be detected in either MZ or FO B cells (data not shown), which confirms a recent study showing low expression of *Mx1* by splenic B cells [13]. These results indicate that prior to disease onset, the TC lupus-prone background leads to an expanded pDC population that accumulates in the MZ and induces a Type I IFN response in MZ B cells.

### Chromatin ICs induce IL-6 and IFN-γ secretion in splenic DCs

**Figure 6:**
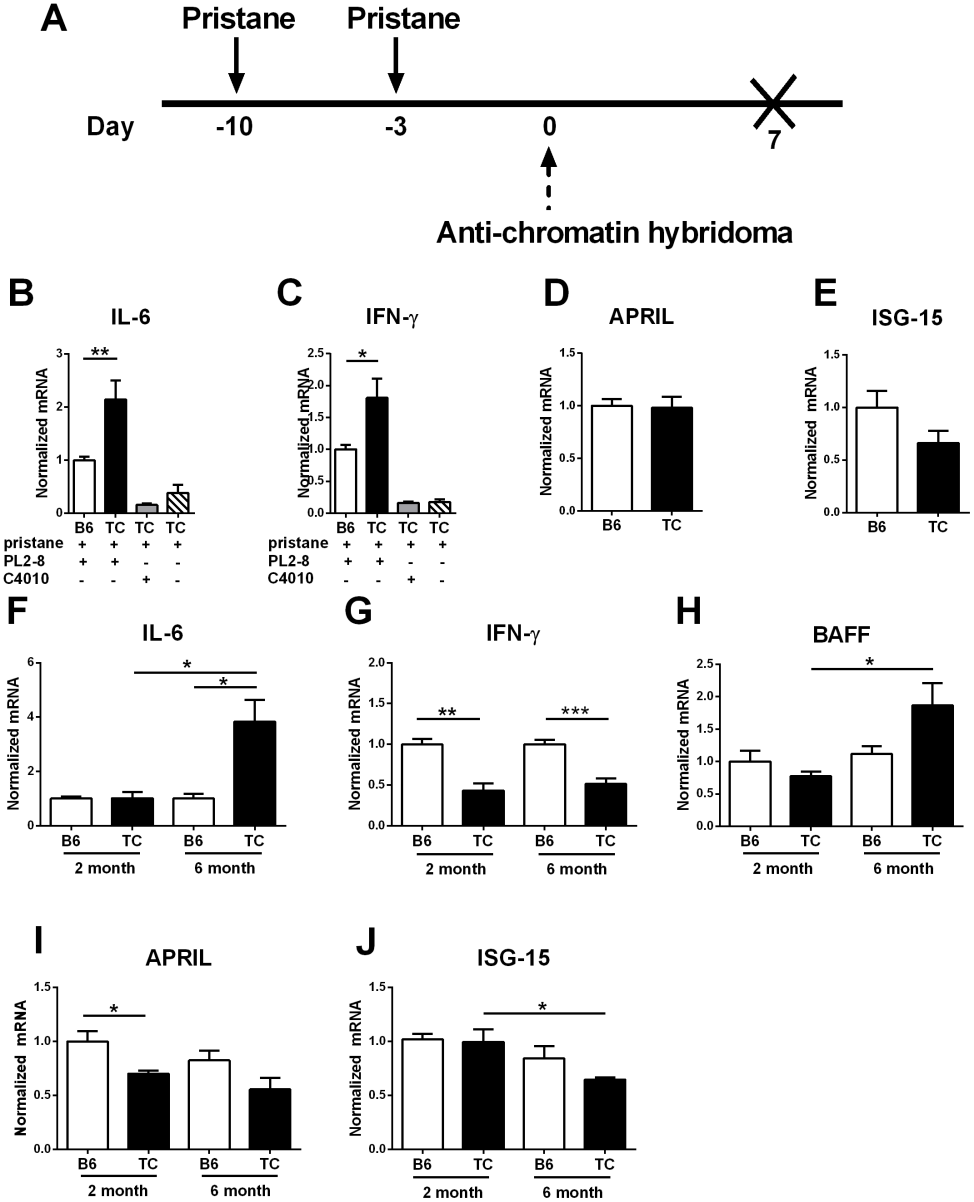
Anti-chromatin ICs induce inflammatory cytokine secretion by splenic DCs in TC mice. **A–E**. Two month old TC and B6 mice were pre-treated with pristane on days -10 and -3 (solid arrows) followed by immunization with an IgG2b anti-chromatin secreting hybridoma (dashed line) on day 0. Anti- anti-TNP IgG2a^b^ C4010 immunization or pristane alone were used as controls. Splenic DCs were isolated on day 7 as shown in **A**, and cytokine expression was quantified by qPCR. Results show the relative mRNA levels of IL-6 (**B**), IFN-γ (**C**), APRIL (**D**), and *Isg-15* (**E**) in these treated mice. **F**–**J**. splenic DCs were obtained from untreated 2 or 6 month old TC or B6 mice and expression levels of IL-6 (**F**), IFN-γ (**G**), BAFF (**H**), APRIL (**I**), and *Isg*-15 (**J**) were quantified by qRT-PCR. Cytokine expression was normalized to *Gapdh* and expressed relative to the mean values for B6 DCs (**B**–**E**) and 2 month old B6 DCs (**F**–**G**). Results are shown as mean and SEM per group (n = 6 per strain). Significance levels of two-tailed t tests are shown.

In addition to Type I IFNs, splenic DCs produce various cytokines in response to TLR7/TLR9 stimulation by anti-chromatin immune complexes (IC) [36], which are a driving factor of lupus pathology [37]. It was of interest to study the DC cytokine response to TLR7/TLR9 ligation *in vivo*, given that TLR7 and TLR9 are expressed at high levels in pDCs [38], a population expanded prior to disease onset in TC mice. To determine the effect of chromatin ICs on the cytokine production by splenic DCs, we treated young B6 and TC mice with PL2-8 hybridoma cells, which secrete IgG2b anti-chromatin Abs that bind endogenous chromatin to form anti-chromatin IgG2b ICs [39]. Mice were pre-treated with pristane to allow for hybridoma cell survival and sacrificed one week after the hybridoma immunization [19], when cytokine levels were quantified from purified splenic DCs (Fig. 6A).

Anti-chromatin ICs increased the production of IL-6 and IFN-γ in both B6 and TC DCs as compared to mice treated with pristane alone or with a hybridoma that does not produce ICs (Fig. 6B–C). IL-6 and IFN-γ production was significantly higher in TC than in B6 DCs. These results showed that anti-chromatin ICs induced a greater production of IL-6 and IFN-γ expression in TC splenic DCs. We also examined the effect of hybridoma immunization on BAFF and APRIL levels. While BAFF expression was undetectable in the splenic DCs from immunized mice, APRIL levels were equal between the two strains (Fig. 6D), confirming the results obtained *in vitro* with stimulated BMDCs. Meanwhile, *Isg-15* has been shown to be expressed at higher levels in B6 pDCs than in TC pDCs [13]. Hybridoma immunization resulted in the same trend, with higher *Isg-15* expression in B6 than in TC splenic DCs (Fig. 6E), which could be due to the fact that anti-chromatin ICs expand the pDC population.

We also compared cytokine expression by splenic DCs from untreated TC and B6 mice. Two-month old TC mice produce very little anti-chromatin IgG, while 6-month old TC mice have produced large amounts of these autoantibodies for at least 2 months. The comparison of the cytokines produced by DCs from these two untreated age groups as well as from age-matched B6 controls therefore provided additional information about TC intrinsic determinants vs. anti-chromatin IC determinants. DCs from the older untreated TC mice showed elevated IL-6 levels (Fig. 6F) but decreased IFN-γ as compared to untreated B6, while there was no difference in the younger mice (Fig. 6G). As the levels of IFN-γ declined with age *in vivo* in TC mice, it is possible that the majority of IFN-γ is secreted by a small subset of DCs whose effects are dependent on location rather than size, but their effect is magnified under strong stimulatory conditions. BAFF expression (Fig. 6H) increased with age in DCs from untreated TC mice, and there was a trend toward a greater expression in older TC than B6 DCs. APRIL levels (Fig. 6I) were lower in untreated TC than B6 DCs and remained unchanged with age. Finally, consistent with the results obtained with anti-chromatin IC immunization, there was a lower mRNA expression of *Isg-15* in untreated older TC mice compared to age-matched B6 (Fig. 6J), which could also be due to the relative expansion of pDCs. These results together suggest that *in vivo* chronic self-antigen or IC stimulation of DCs mainly induced IL-6 in TC mice.

### Apoptotic cells preferentially reduce IL-6 secretion in B6 MZ B cells

**Figure 7:**
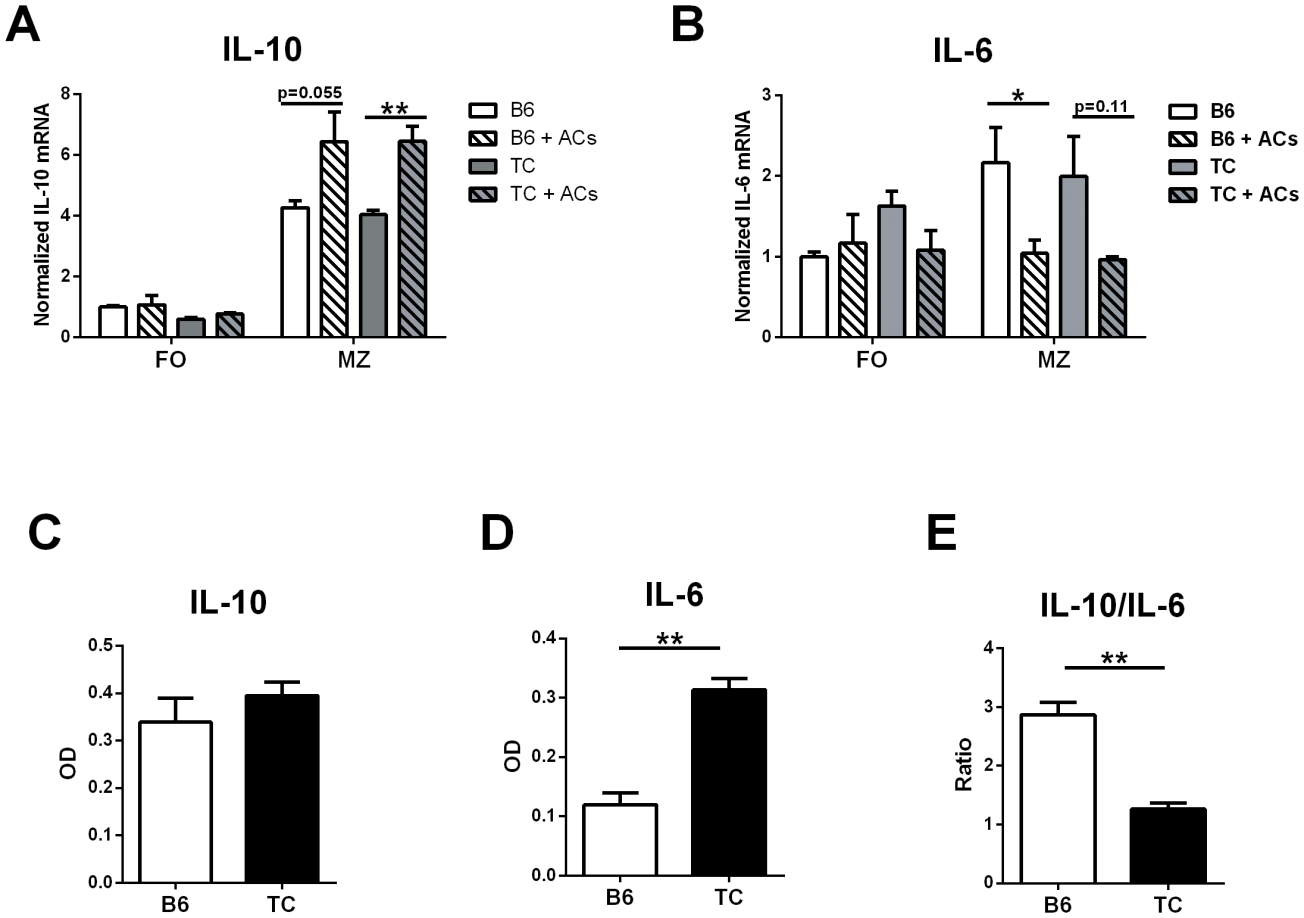
Apoptotic cells preferentially reduce IL-6 secretion by B6 but not TC MZ B cells. Sorted FO and MZ B cells (N = 3 per strain from 2 different sorts) were cultured for 3 d with CpG in the presence or absence of apoptotic cells. IL-10 (**A**) and IL-6 (**B**) mRNA levels in MZ and FO B cells were quantified by qRT-PCR. Message expression was normalized to *Gapdh* and expressed relative to B6 FO B cells. Data from three independent experiments are shown. Culture supernatant from TC and B6 MZ B cells cultured with apoptotic cells was assayed for IL-10 (**C**) and IL-6 (**D**) by ELISA. **E**. Ratio of IL-10 to IL-6 production of MZ B cells cultured with apoptotic cells. Results show mean and SEM value. Significance levels of two-tailed t-tests are shown.

Anti-chromatin ICs also have the potential to elicit a direct response from B cells given that they also express TLR7 and TLR9. Furthermore, TLR9 signaling is essential for the production of the anti-inflammatory cytokine IL-10 by B cells when exposed to apoptotic cells [40]. We hypothesized that the production of inflammatory cytokines by DCs from TC mice impaired the anti-inflammatory response of B cells in response to apoptotic cells. We tested this hypothesis by comparing the down-regulation of IL-6 and up-regulation of IL-10 expression by B6 and TC B cells in response to apoptotic cells. Expression of IL-10 can be induced at high levels in MZ, but not FO, B cells co-cultured with apoptotic cells and this upregulation was associated with the maintenance of tolerance in normal mice [40]. Therefore, splenic B cells from B6 and TC mice were sorted into FO and MZ B cells and co-cultured with B6 apoptotic cells. This resulted, as expected, by the production of IL-10 by MZ, but not FO, B cells (Fig. 7A). B6 and TC MZ B cells had similar levels of IL-10 mRNA following induction. Interestingly the increased IL-10 levels coincided with significantly decreased IL-6 levels in B6 but the difference was not significant for TC MZ B cells (Fig. 7B).

To further understand if the IL-6 down-regulation in the presence of IL-10 is impaired in the B cells from lupus-prone TC mice, we measured the cytokine levels in the culture supernatants. Confirming the qPCR results, IL-10 levels were equal between B6 and TC MZ B cells cultured with apoptotic cells (Fig. 7C). However, TC MZ B cells secreted significantly more IL-6 than B6 MZ B cells (Fig. 7D). Therefore, the IL-10 to IL-6 ratio was higher in B6 MZ B cells (Fig. 7E). This indicates that the TC lupus-prone B cells are less responsive to anti-inflammatory conditions, resulting in the inefficient down-regulation of the inflammatory cytokine IL-6.

## Discussion

B cell tolerance is dysregulated in SLE resulting in the secretion of pathogenic autoAbs. Cytokines play an important role in both promoting and regulating immune responses. We have previously shown that activated DCs promote B cell proliferation and Ab secretion without direct cell to cell interaction via the secretion of cytokines [3]. Furthermore, TC DCs elicit a stronger B cell response than B6 DCs. Here we show that both IL-6 and IFN-γ are required for the TC DC-mediated enhancement of B cell proliferation. However, IL-6 and IFN-γ can compensate for each other as dual inhibition was necessary to restore proliferation to normal levels. Meanwhile, inhibition studies showed that IL-6, but not IFN-γ, was essential for inducing IgM secretion in both B6 and TC mice.

TC DCs did not promote B cell proliferation through the secretion of B cell survival factors as BAFF and APRIL secretion was not elevated in anti-CD40 activated TC DCs. Although unstimulated TC BMDCs produce significantly more BAFF and APRIL than B6 BMDCS, anti-CD40 stimulation eliminated this difference. Given that similar results were obtained after *in vivo* TLR stimulation with anti-chromatin ICs, this suggests that DC-derived BAFF and APRIL do not play a significant role in the dysregulation of TC B cells in inflammatory conditions. Since BAFF levels are elevated in NZM2410 mice [26] and in SLE patients [41], it is possible that BMDCs before they are activated in the periphery by T cells or nucleic acid-containing ICs or other cell types are responsible for regulating B cells via the secretion of BAFF and APRIL survival factors. Therefore, activated TC DCs promote B cell proliferation and Ab secretion exclusively by the secretion of pro-inflammatory cytokines.

The recent report showing that Blimp-1 deficiency in DCs resulted in a lupus phenotype that was dependent on IL-6 production [27] led us to investigate whether the high level of IL-6 production by TC DCs was due to a Blimp-1 deficiency. Moreover, PRDM1/BLIMP-1 polymorphisms have been associated with SLE susceptibility in genome-wide association studies [42]. Our results showed, however, a significantly higher Blimp-1 expression in TC BMDCs than in B6, which remained higher after anti-CD40 stimulation. Blimp-1 negatively regulates DC development but positively regulate DC maturation [43]. More specifically, Blimp-1 expression increases in DCs after TLR activation in a p38 MAPK and NF-kB-dependent manner. Therefore, the increase in Blimp-1 expression in TC DCs fits with their increased level of activation and function [25]. Our results showed, however, that the increased IL-6 production occurs in spite of high Blimp-1 level in B6.TC mice.

pDCs have been a main focus of DC studies in SLE given their high secretion of Type-I IFNs after TLR7/TLR9 stimulation [10]. A previous study showed that both pDCs and myeloid DCs are expanded in aged TC mice [25]. Furthermore, TC DCs express a Type I IFN signature prior to disease onset [13]. We have not been able to reproduce this finding for BMDCs from young mice, and at this time it is not clear whether different environmental conditions for the mice or experimental conditions for the DCs are responsible for this discrepancy. However, we showed that TC mice have an expanded pDC population prior to disease onset while the myeloid and lymphoid subsets increase with disease progression. Interestingly, we found that the pDCs were enriched in the MZ in both B6 and TC mice, but this expansion does not overlap with the DCIR2^+^ subset that has been recently reported in the MZ [35]. pDCs expansion and localization to the MZ has been previously reported after CpG treatment [44], Toxoplasma infection [45], as well as primary cutaneous marginal zone lymphomas [46]. Our study is the first, to our knowledge, to report a naturally occurring concentration of pDCs in the MZ that expands with age in autoimmune mice. Accordingly, MZB cells displayed an enhanced type I IFN signature as compared to FO B cells, and there was a significant difference between the TC and B6 mice. We have shown that type I IFN-activated MZB cells promote autoantigen transport and induce T cell activation in BXD2 lupus-prone mice [47], [48]. Our present results suggest that the local activation of MZB cells by pDCs is likely a critical event that occurs in lupus pathogenesis.

The TLR7/TLR9 signaling pathway is complex and results in the secretion of different pro-inflammatory cytokines depending on the structure and location of the TLR ligands [49], [50]. Therefore, TLR7/TLR9 signaling can also affect pathology in a Type-I IFN independent manner. DCs that lacked the TLR-adaptor protein MyD88 showed an overall decrease in the levels of inflammatory cytokines in the spleen after TLR9 stimulation with CpG [36], [51]. Furthermore, TLR signaling by DCs also affects B cell function as MyD88 deficient DCs had significantly lower levels of IgG2a anti-nucleosome autoAbs [52]. In addition, human pDCs stimulated with ICs isolated from SLE patients induced greater levels of inflammatory cytokines, including IL-6 and IFN-γ, in a TLR-9 dependent manner [53]. Here we showed that *in vivo* exogenous administration of anti-chromatin ICs resulted in elevated production of IL-6 and IFN-γ by splenic DCs of TC mice. Thus TLR7/9 stimulation promoted secretion of pro-inflammatory cytokines in lupus prone mice, potentially as a result on the expansion MZ pDCs.

Given the high levels of pro-inflammatory cytokines found in the TC lupus background, it is difficult to determine if the expression of anti-inflammatory cytokines is altered in these mice. We focused on the expression of IL-10, an anti-inflammatory cytokine that has been proposed to both contribute to and protect from SLE. SLE patients present elevated levels of serum IL-10 that correlate with disease activity [54], and polymorphisms in the IL-10 gene have been associated with SLE susceptibility [55], [56], with the risk allele associated with an increased expression [57]. On the other hand, IL-10 overexpression on the TC background reduced total IgM and delayed the production of ANAs [58]. Furthermore, IL-10 has suppressive effects when produced by regulatory B cells (Breg) [59], and the loss of IL-10 production by these B cells results in systemic autoimmunity [60]. Finally, the clearance of apoptotic cells requires the production of IL-10 to prevent the induction of autoimmunity [61]. Following the experimental protocol by Miles et al [62], we induced IL-10 secretion in MZ B cells co-cultured with apoptotic cells Under these conditions TC MZ B cells expressed equal mRNA levels of IL-10 than B6 MZ B cells and in both strains this treatments resulted in decreased levels of IL-6. Interestingly, cytokine quantification from the culture supernatant showed that TC mice still had significantly higher expression of IL-6 under anti-inflammatory conditions and thus the IL-10 to IL-6 ratio for these mice was lower than for B6. These results indicate that although IL-10 secretion is equally induced in both strains, the lupus-prone TC mice do not effectively reduce IL-6 levels under anti-inflammatory conditions.

In summary, the cytokine milieu in TC lupus background favors the production of pro-inflammatory cytokines capable of modulating B cell responses. In particular, IL-6 and IFN-γ secretion by TC DCs enhances B cell proliferation. TC mice have an expanded pDC population that localizes to the MZ and correlates with an elevated Type I IFN response in MZ but not FO B cells. In addition, TLR7/TLR9 stimulation resulted in the secretion of higher levels of IL-6 and IFN-γ by splenic DCs. However, under anti-inflammatory conditions the TC lupus background cannot efficiently repress production of IL-6 by MZ B cells. Taken together, dysregulations in cytokine networks are present in the lupus-prone TC mice and aid in the breakdown of B cell tolerance.

## Supporting Information

Figure S1 **Pathway analysis of gene expression in B6 B cells cultured with supernatant from anti-CD40 stimulated BMDCS from either B6 or TC mice.** Green and red symbols show genes significantly over-expressed in B cells exposed to B6 and TC-produced BMDC supernatant, respectively. White symbols show genes that represent functional intermediates in the pathways.
(PPTX)
