# Supplementary figures and images for: Dysregulated Cytokine Production by Dendritic Cells Modulates B Cell Responses in the NZM2410 Mouse Model of Lupus
Source: PLoS One. 2014 Aug 5;9(8):e102151. doi: 10.1371/journal.pone.0102151 (PMC4122346; doi:10.1371/journal.pone.0102151)

## Slide 1
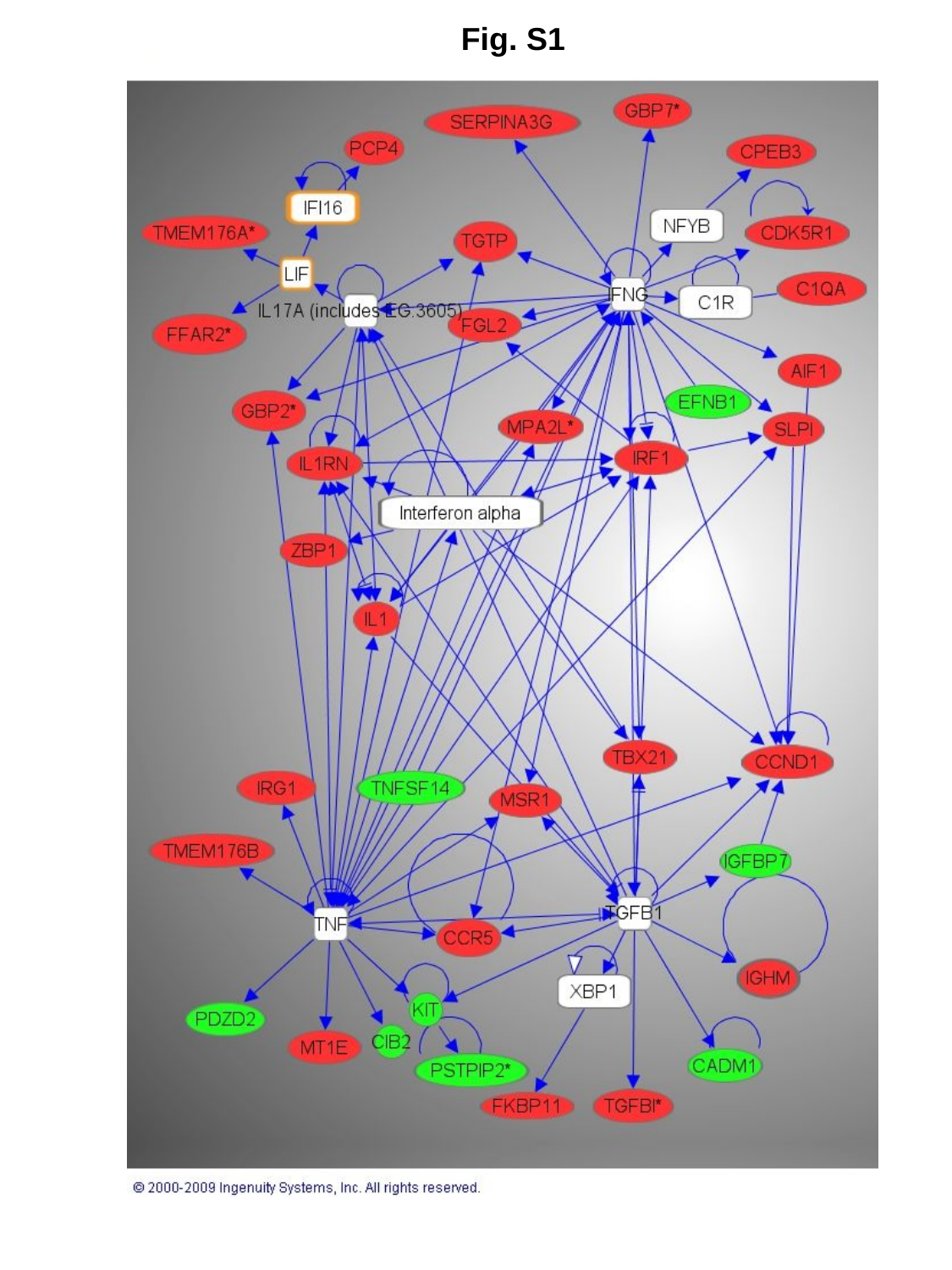

Fig. S1

Supplement: Figure S1 — Pathway analysis of gene expression in B6 B cells cultured with supernatant from anti-CD40 stimulated BMDCS from either B6 or TC mice. Green and red symbols show genes significantly over-expressed in B cells exposed to B6 and TC-produced BMDC supernatant, respectively. White symbols show genes that represent functional intermediates in the pathways. (PPTX) [file pone.0102151.s001.pptx]
